# Sensitivity and specificity of magnetic resonance imaging and computed tomography for the determination of the developmental state of cranial sutures and synchondroses in the dog

**DOI:** 10.1186/s12917-019-1967-9

**Published:** 2019-07-01

**Authors:** Daniela Farke, Carsten Staszyk, Klaus Failing, Robert M. Kirberger, Martin J. Schmidt

**Affiliations:** 1 Department of Veterinary Clinical Sciences, Small Animal Clinic, Justus-Liebig-University, Frankfurter Strasse 108, 35392 Giessen, Germany; 2 Institute of Veterinary-Anatomy, -Histology, and –Embryology, Justus-Liebig-University, Frankfurter Strasse 98, 35392 Giessen, Germany; 3 Department of Biomathematics, Justus-Liebig-University, Frankfurter Strasse 95, 35392 Giessen, Germany; 4 Department of Companion Animal Clinical Studies, Faculty of Veterinary Science, University of Pretoria, Private Bag X04, Onderstepoort, Pretoria, 0110 South Africa

**Keywords:** Brachycephaly, Canine, Craniosynostosis, Osteogenesis, Skull

## Abstract

**Background:**

During skull ontogenesis, growth centers in the skull base and calvarial bones allow gradual expansion of the cranial vault. Premature growth termination of cranial base synchondroses and/or calvarial sutures can result in devastating skull dysmorphologies. There is evidence to believe that a premature closure in one or more cranial growth centers contribute to the brachycephalic skull morphology in dogs. To provide a proof of concept for the non-invasive investigation of ontogenetic changes in cranial sutures and synchondroses in living dogs, we compared magnet resonance imaging (MRI) and computed tomography (CT) with histologic findings. Our aim was to determine the in vitro sensitivity and specificity for conventional clinical imaging methods in the assessment of cranial suture closure and synchondroses ossification in dogs.

**Results:**

The evaluation of cranial base synchondroses in MRI had a sensitivity of up to 93.1% and a specificity of 72.7% dependent on the observer. The evaluation of cranial base synchondroses in CT had a sensitivity of 92.2% and a specificity of 86.4%. Suture assessment on MRI suture assessment had a sensitivity of 82.1% dependent on the observer and a specificity of 19.3%. CT suture assessment had a sensitivity of 85.1% and a specificity of 40.4% in dependence of the observer.

**Conclusion:**

Conventional cross-sectional imaging techniques (MRI and CT) allow reliable assessment of the open or closed state of synchondroses within the cranial base. In contrast CT and MRI are not suitable for a reliable assessment of the cranial sutures in dogs.

## Background

The mammalian skull develops as a composite structure that consists of a multitude of tissues originating from different embryonic precursors [1]. The dynamic development to a solid braincase occurs in synchrony, and in coordination with the underlying brain [2]. During fetal development, separate mineralization centers arise in the membranous calvarial primordium that covers the dorso-lateral brain.These ossification centers gradually expand and give rise to the paired parietal-, frontal-, temporal-, and the unpaired supraoccipital, ethmoidal- and interparietal bones (membranous ossification). With increased growth, the opposing borders of the cranial bone primordia meet, forming thin areas with sustained osteo-proliferative capacity called cranial sutures [3, 4]. New bone is gradually formed at the edges of the bone fronts allowing postnatal expansion of the skull vault in synchrony with brain expansion [5].

Contrary to the calvaria, the bones of the cranium base develop by enchondral ossification [3, 6, 7]. The basioccipital, basisphenoid and presphenoid bones emerge from cartilaginous precursors that ossify during fetal development. Cartilaginous segments termed synchondroses persist between the ossification centers, consisting of two mirror-image growth plates arranged in opposing directions. Analogous to endochondral growth plates in long bones, synchondroses of the skull base grow through ongoing chondrocyte proliferation and gradual osseous transformation allowing the expansion of the cranial base along its rostro-caudal axis [1, 2, 8].

The growth of individual bones is regulated within these centers by complex signal cascades, involving multiple receptors and transcription factors within both kinds of growth centers [9]. Any disturbance in these processes has been shown in children to lead to premature closure of both, cranial base synchondroses and/or sutures (craniosynostoses) and thereby to devastating skull dysmorphologies [10]. Regulation of growth and closure of both, sutures and synchondroses of the skull have been extensively studies in laboratory rodents [11, 12], but not so in dogs. Even the knowledge of general temporary evolution of skull sutures and synchondroses in companion animals is limited. Two older investigations determined closure times for the cranium growth centers in dogs at the age of 12 months [13, 14]. Two recent studies documented a higher incidence of closed facial sutures and premature closure times for the spheno-occipital synchondrosis in brachycephalic dogs compared to mesaticephalic dogs [15, 16]. The authors of both studies suggest that the temporal variation of growth termination has a substantial influence for the development of a brachycephalic head morphology. In light of these new insights, the question arises as to whether these variations are part of a physiologic spectrum or, at least partially, a pathological condition. It should be considered that different forms of pathologic craniosynostoses and grades might contribute to the brachycephalic skull morphology in dogs in the same way as it does in cats [17]. Breeding of phenotypes based on pathological genetic defects clearly known to be associated with neurological or craniofacial diseases as in humans [18] would be prohibited by the German animal protection law and would also be unethical.

Cross sectional diagnostic imaging methods (MRI and CT) would allow the examination of large cohorts of dogs of different breeds in vivo*.* However, the value of these imaging methods for the assessment of synchondrosal and sutural status has never been determined.

The aim of the present study was, therefore, to compare imaging findings with histological preparations in order to determine the sensitivity and specificity for conventional clinical CT and MRI in the assessment of the open or closed status of cranial synchondroses and sutures in dogs.

## Results

### Histology

Due to the fragility of the structures, especially in very young puppies, twenty-two sutures and five synchondroses were lost during sample preparation. 433 sutures and 112 synchondroses could be histologically evaluated.

#### Synchondroses morphology and status

**Fig. 1:**
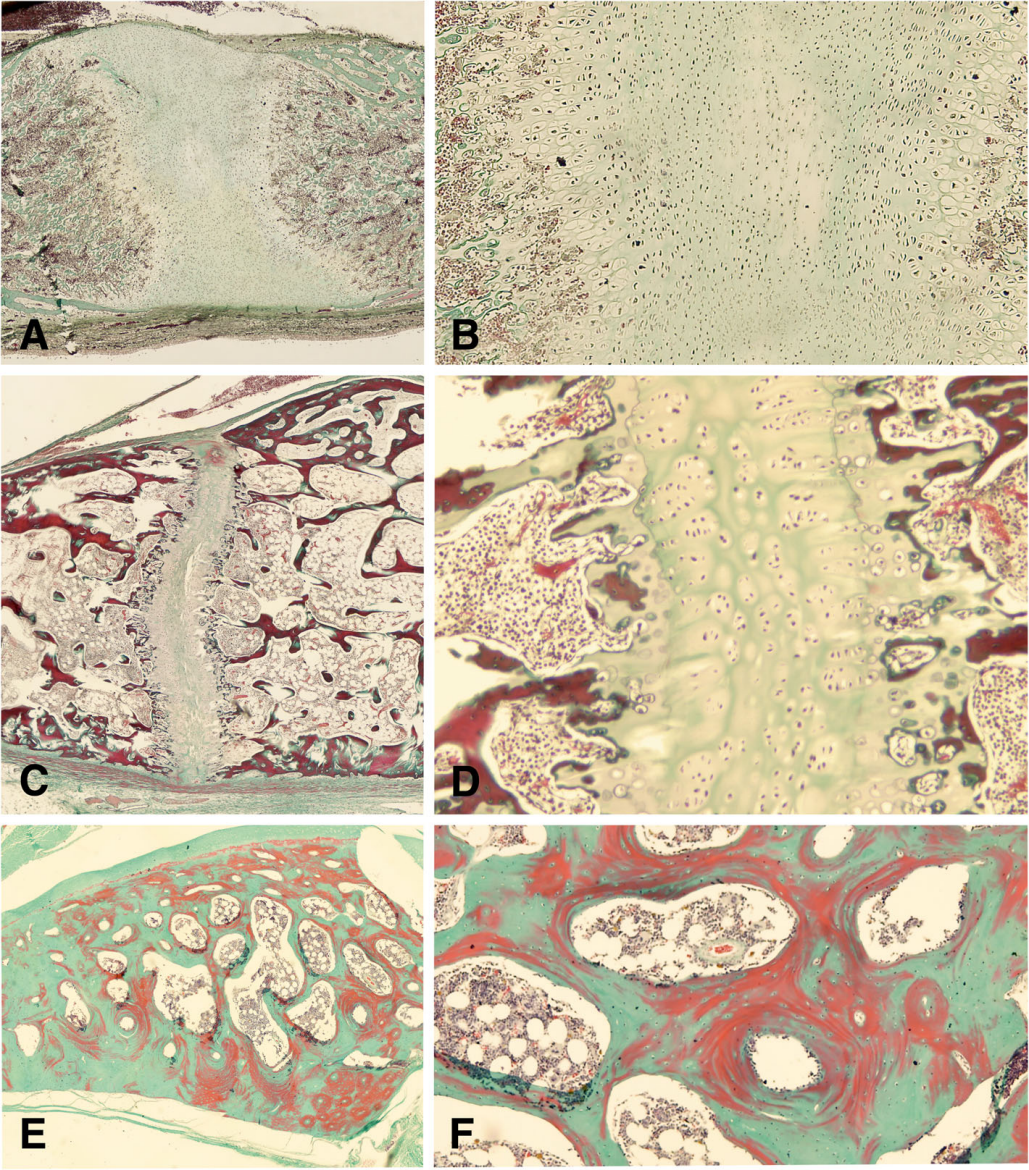
Histological sections of skull base synchondroses in different stages. Photomicrographs of Masson Goldner-trichrome stained histologic sections through the skull base of a 3 days old Pyrennean shepherd dog (**a**, **b**), a 5 months old Shiba Inu (**c**, **d**), and a 3 years old German Shepherd dog (**e**, **f**) in overview (**a**, **c**, **e**) and 40 x magnification (**b**, **d**, **f**), demonstrating the temporal evolution of the skull spheno-occipital synchonrdrosis

In all immature dogs, a double-sided arrangement of chondrocytes in a hyaline homogenous cartilage matrix was observed between the bone tissue of the basicranial bones. The cartilage consisted of chondrocytes being distributed into a central resting zone, as well as bilateral proliferating, and hypertrophic zones (Fig. 1 A, B) [19]. In the fourth zone in the peripheral portion of synchondrosis, osteoblasts and blood vessels invaded the area of cartilage. In older dogs, the resting and proliferative layer within the synchondrosis gradually decreased, leading to a relative narrowing of the growth center (Fig. 1c and d). At the chondro-osseous junction differentiated hypertrophic chondrocytes are replaced by bony trabecula that, eventually, fill the growth plate leading to a continuous medullary cavity (Fig. 1e and f).

90 synchondroses (80.4%) were evaluated as closed, and 22 (19.6%) were assessed as open. The spheno-occipital synchondroses were classified as open in 31 (83.78%) specimen as closed in 6 (16.22%). Of the 38 examined inter-sphenoidal synchondroses, 32 (84.21%) were classified as open, and 6 (15.79%) as closed. Of 37 examined spheno-ethmoidal synchondroses, 27 (72.97%) were classified as open and 10 (27.03%) as closed.

#### Suture morphology and status

**Fig. 2:**
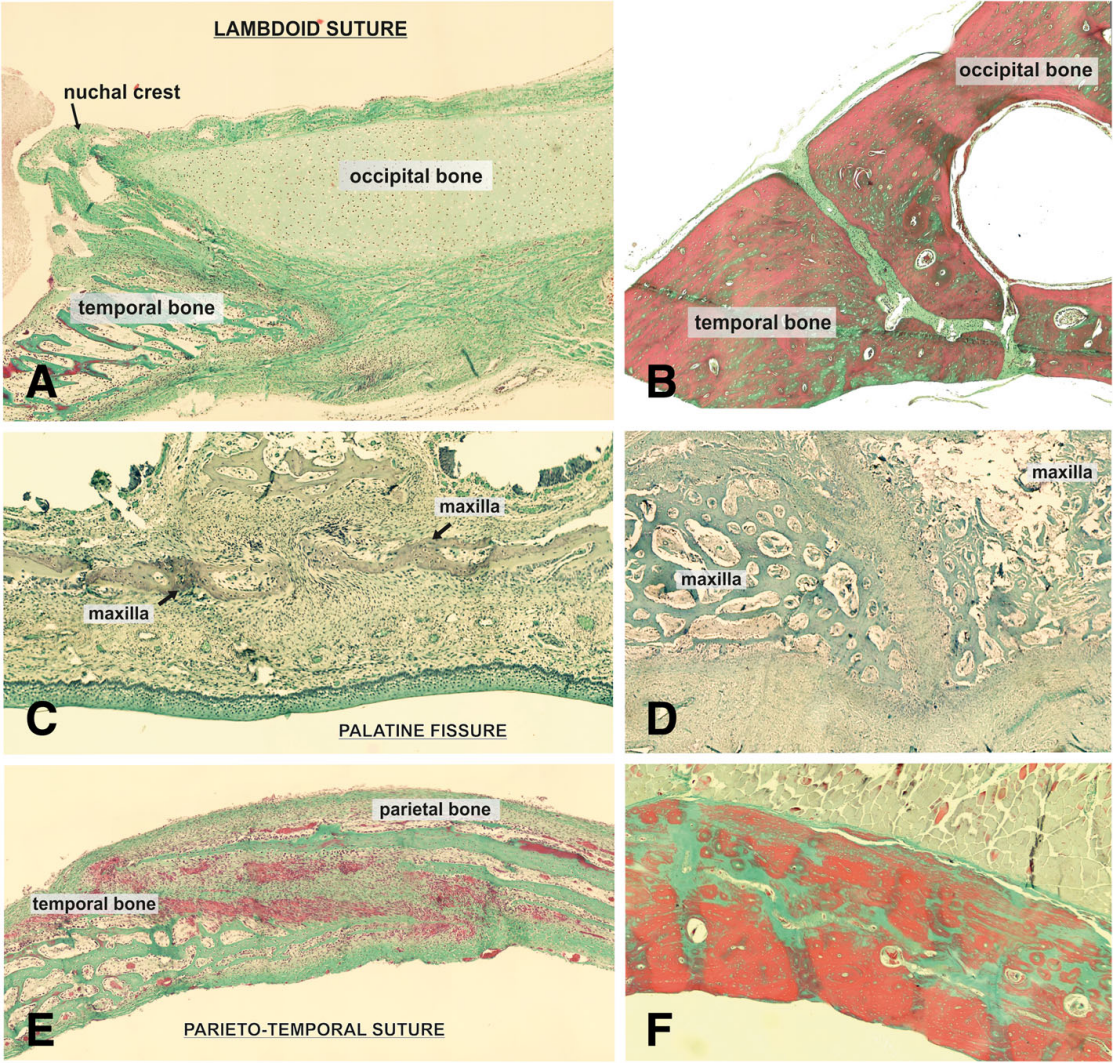
Histological sections of cranial sutures in different stages. Photomicrographs of Masson Goldner-trichrome stained histologic sections through the lambdoid suture (**a**,**b**) the palatine fissure (**c**, **d**) and the parieto-temporal suture (**e**, **f**) of a 3 days old Pyrenean shepherd dog (**a**, **c**, **e**), and an 11 years old Spanish greyhound (**b**, **d**, **f**)

**Fig. 3:**
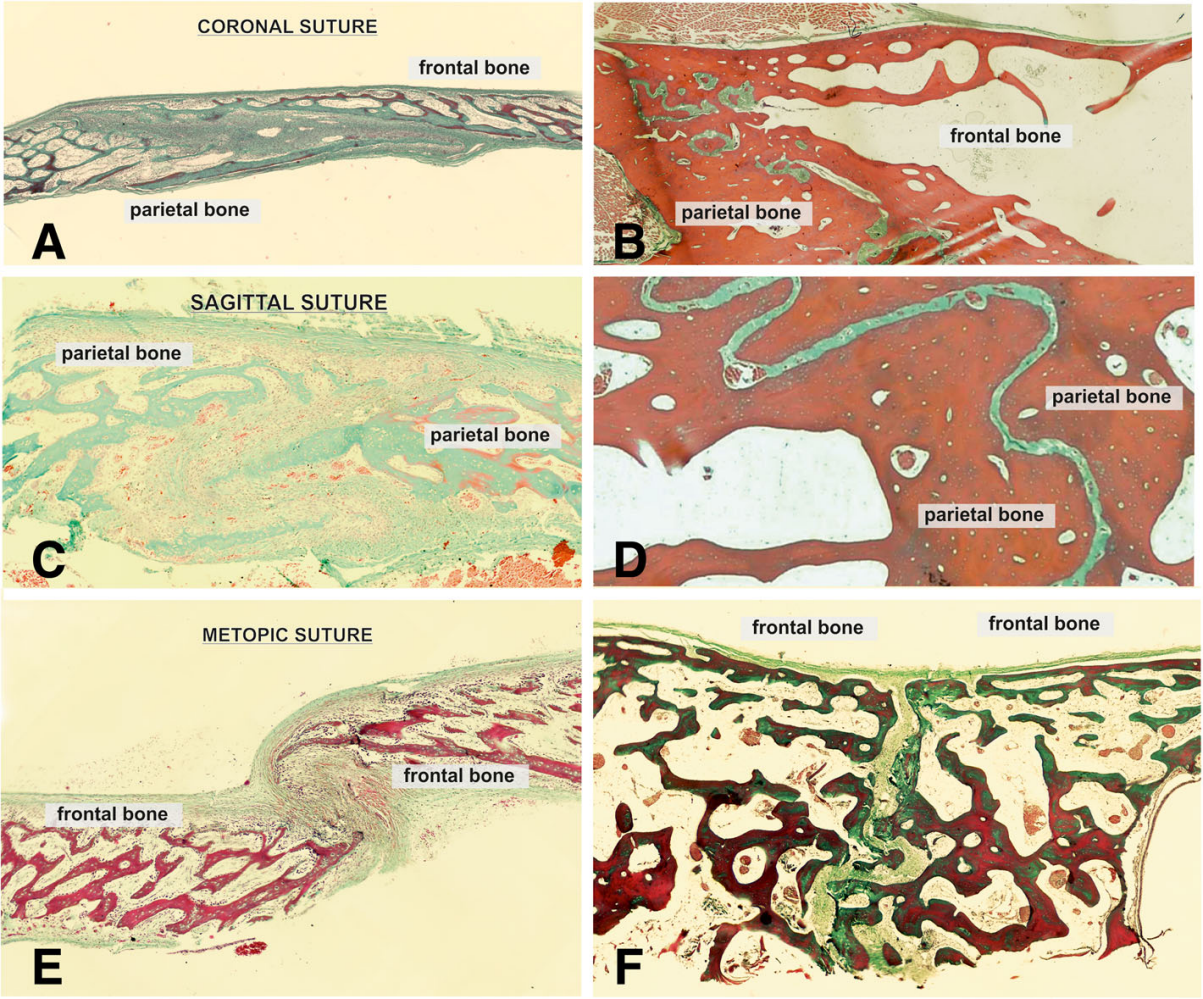
Histological sections of cranial sutures in different stages. Photomicrographs of Masson Goldner-trichrome stained histologic sections through the coronal suture (**a**,**b**) the sagittal (**c**, **d**) and the metopic suture (**e**, **f**) of a 3 days old Pyrenean shepherd dog (**a**, **c**, **e**), and an 11 years old Spanish greyhound (**b**, **d**, **f**)

**Fig. 4:**
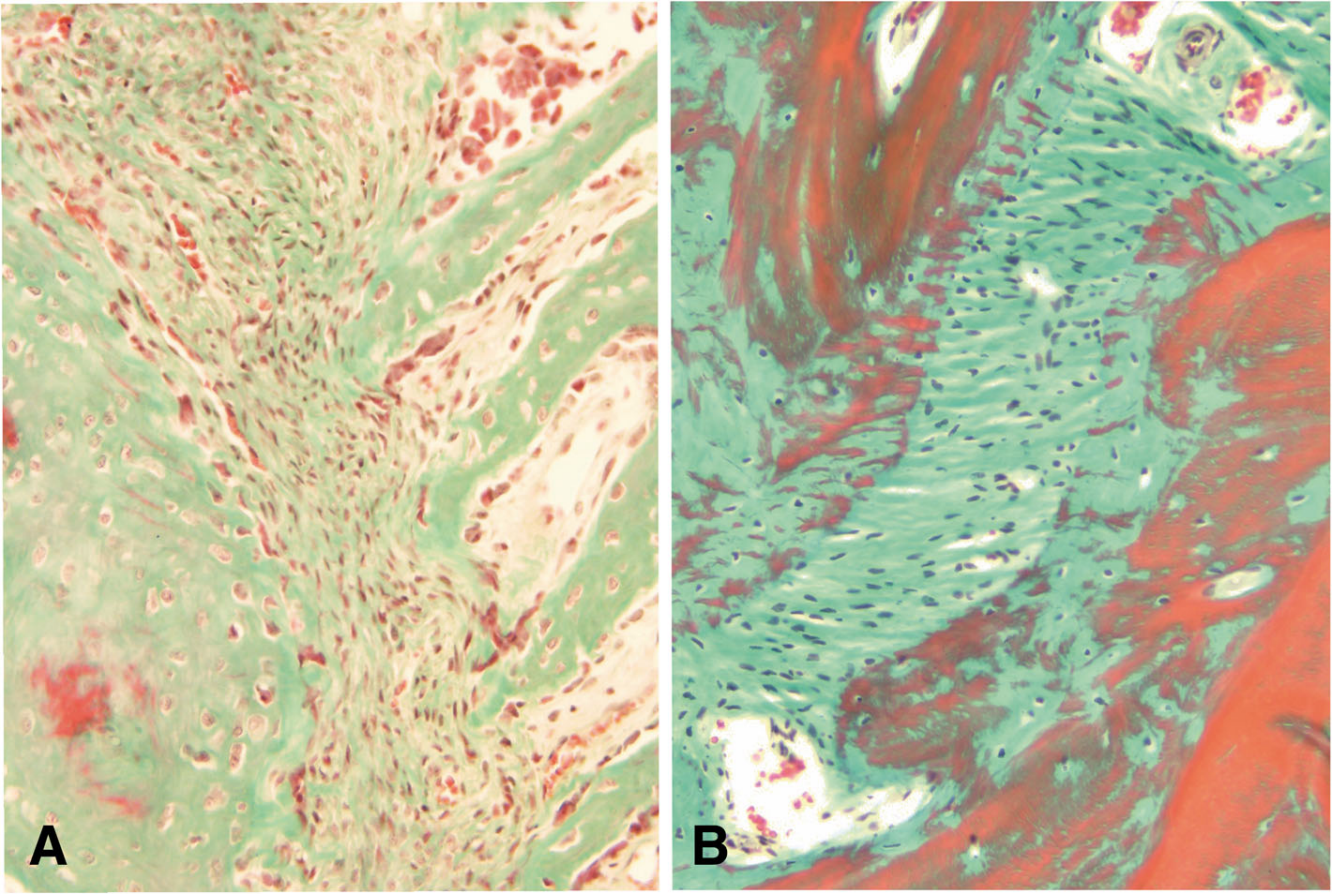
Histological sections of cranial sutures in different stages. Photomicrographs of Masson Goldner-trichrome stained histologic sections through the sagittal suture of a 3 days old Pyrenean shepherd dog (**a**), and an 11 years old Spanish greyhound (**b**)

Suture morphology ranged from straight-edged plane sutures (lambda suture; Fig. 2 A, B) or butt-sutures (palatine fissure, Fig. 2c and d) over simple overlapping (sphenofrontal and squamosal suture, Fig. 2e and f; coronal suture, Fig. 3a and b) to serrated sutures, with the bone edges having a saw-like appearance (sagittal suture, Fig. 3c and d). The gap in between the bony edges are filled with collagen and elongated fibrocytes. Two types of open sutures were identified according to the type of connective tissue, and cellular components, which dominated the sutural space. In young dogs the sutures contained loosely arranged connective tissue showing a collagen fiber orientation preferentially parallel to the sutural alignment (Fig. 4a). These sutures also featured high amounts osteoblasts (> 50 in a mean of 3 FOV) and fibroblasts (> 20 in 3 FOV) (type A; Fig. 4a). In older dogs (> 7 months), sutures rather contained a more dense connective tissue with collagen fiber orientation being orientated oblique to perpendicular to the suture line, with a low numbers of osteoblasts (< 10 in 3 FOV) and fibroblasts (< 20 in 3 FOV) (type B, Fig. 4b).

From 433 sutures, 240 (55.4%) of the sutures were categorized as open type A syndesmoses, 78 (18%) were classified as type B syndesmoses (open). No suture line was observed in 115 (26%) of the examined sutures.

### Sensitivity and specificity of synchondrosis assessment using MRI

In dependence of the observer, 87 (inexperienced observer, DF) - 89 (experienced observer, MS) synchondroses were classified as open, and 22 as closed. For all synchondroses there was a high accordance of MRI and histological findings. A sensitivity of 93.3% (confidence level 95% of 77.9–99.9%) and specificity of 83.3–100% (confidence level 95% of 35.9–100%) was found for the spheno-occipital synchondrosis. A high sensitivity of 93.5% (CI 95% of 78.6–99.2%) and low to moderate specificity of 50 (MS) - 66.7% (DF) (CI 95% of 11.8–95.7%) for intersphenoidal synchondrosis. A high sensitivity of 84.6 (DF) - 92.3% (MS) (CI 95% of 65.1–95.6%) and a moderate specificity of 70% (CI 95% of 34.8–93.3%) was shown for spheno-ethmoidal synchondrosis (Table 1).

**Table 1:** MRI sensitivity and specificity for sutural and synchondrosal assessement in both observers. Observer 1 (inexperienced) and observer 2 (experienced)

Suture/Synchnondrosis	Observer 1 sensitivity	Confidence level > 95%	Observer 1 specificity	Confidence level > 95%	Observer 2 sensitivity	Confidence level > 95%	Observer 2 specificity	Confidence level > 95%
S1	87.5%	67.6–97.3%	15.4%	1.9–45.4%	95.8%	78.9–99.9%	15.4%	1.9–45.4%
S2	76.9%	56.4–91.0%	25%	5.5–57.2%	80.8%	60.6–93.4%	25%	5.5–57.2%
S3	73.9%	51.6–89.8%	14.3%	1.8–42.8%	78.3%	56.3–92.5%	21.4%	4.7–50.8%
S4	88%	86.8–97.5%	16.7%	2.1–48.4%	84%	63.9–95.5%	16.7%	2.1–48.4%
S5	61.6%	35.7–82.7%	18.8%	4.0–45.6%	77.8%	52.4–93.6%	31.3%	11.0–58.7%
S6	52.2%	30.6–73.2%	15.4%	1.9–45.4%	69.9%	47.1–86.8%	23.1%	5.0–53.8%
S7	70%	45.7–88.1%	18.8	4.0–45.6%	85%	62.1–96.8%	31.3%	11.0–58.7%
S8	73.1%	52–2-88.4%	16.7%	2.1–48.4%	80.8%	60.6–93.4%	25%	5.5–57.2%
S9	66.7%	41.0–86.7%	11.8%	1.5–36.4%	77.8%	52.4–93.6%	17.6%	3.8–43.4%
S10	66.7%	22.3–95.7%	3.8%	0.1–19.6%	100%	60.7–100%	7.7%	0.1–25.1%
S11	50%	15.7–84.3%	4.0%	0.1–20.4%	100%	68.8–100%	8.0%	0.1–26.0%
S12	76.5%	50.1–93.2%	33.3%	0.1–65.1%	76.5%	50.1–93.2%	27.3%	6.0–61.0%
SO	93.3%	77.9–99.2%	83.3%	35.9–99.6%	93.3%	77.9–99.2%	100%	54.1–100%
IS	93.5%	78.6–99.2%	66.7%	22.3–95.7%	93.5%	78.6–99.2%	50%	11.8–88.2%
SE	84.6%	65.1–95.6%	70%	34.8–93.3%	92.3%	74.9–99.1%	70%	34.8–93.3%

**Table 2:** CT sensitivity and specificity for sutural and synchondrosal assessement in both observers. Observer 1 (inexperienced) and observer 2 (experienced)

Suture/Synchnondrosis	Observer 1 sensitivity	Confidence level > 95%	Observer 1 specificity	Confidence level > 95%	Observer 2 sensitivity	Confidence level > 95%	Observer 2 specificity	Confidence level > 95%
S1	83.3%	62.6–95.3%	35.7%	12.8–64.9%	87.5%	67.6–97.3%	28.6%	8.4–58.1%
S2	80.8%	60.6–93.4%	69.2%	38.6–90.9%	84.6%	65.1–95.6%	76.9%	46.2–95.0%
S3	83.3%	62.6–95.2%	71.4%	41.9–91.6%	83.3%	62.6–95.2%	71.4%	41.9–91.6%
S4	76.9%	56.4–91.0%	41.3%	15.2–72.3%	88.5%	69.8–97.6%	25%	5.5–57.2%
S5	73.7%	48.8–90.9%	43.7%	19.8–70.1%	78.9%	54.4–93.9%	43.7%	19.8–70.1%
S6	75%	53.3–90.2%	46.2%	19.2–74.9%	75%	53.3–90.2%	46.2%	19.2–74.9%
S7	85.7%	63.7–97.0%	50%	24.7–75.3%	85.7%	63.7–97.0%	50%	24.7–75.3%
S8	81.5%	61.9–93.7%	50%	21.1–78.9%	81.5%	61.9–93.7%	50%	21.1–78.9%
S9	77.8%	52.4–93.6%	27.8%	9.7–53.5%	83.3%	58.6–96.4%	22.2%	6.4–47.6%
S10	83.3%	35.8–99.6%	25.9%	11.1–46.3%	100%	60.7–100%	22.2%	8.6–42.3%
S11	87.5%	47.3–99.7%	26.9%	11.6–47.8%	100%	68.8–100%	23.1%	9.0–43.6%
S12	100%	83–8-100%	25%	5.5–57.2%	94.4%	72.7–99.9%	25%	5.5–57.2%
SO	96.8%	83.3–99.9%	100%	54.1–100%	96.8%	83.3–99.9%	100%	60.7–100%
IS	93.7%	79.2–99.2%	83.3%	35.9–99.6%	93.7%	79.2–99.2%	83.3%	35.9–99.6%
SE	85.2%	66.3–95.8%	80%	44.4–97.5%	85.2%	66.3–95.8%	80%	44.4–97.5%

### Sensitivity and specificity of synchondrosis assessment using CT

In CT evaluation of the skulls 84 (DF)-85 (MS) synchondroses were determined as open. In 91.1–93.3% of the cases (CI 95% of 84.2–95.6%) synchondroses were identified correctly as open. Closed synchondroses were correctly identified in 86.4% of the samples (CI 95%, 65.1–97.1%). The spheno-ocipital synchondrosis showed the highest sensitivity of 96.8% (CI 95% of 83.3–99.9%) and highest specificity of 100% (CI 95% of 60.7–100%) The intersphenoidal synchondrosis showed a high sensitivity of 93.7% (CI 95% of 79.2–99.2%) and specificity of 83.3% (CI 95% of 35.9–99.6%). The lowest accordance of CT and histological findings was shown with a high sensitivity of 85.2% (CI 95% 66.3–95.8%) and a high specificity of 80% (CI 95% of 44.4–97.5%) in the spheno-ethmoidal synchondrosis (Table 2).

### Sensitivity and specificity of suture assessment using MRI

MRI suture assessment shows a moderate to high sensitivity of 72.2 (DF) -82.1% (MS) (CI 95% of 66.0–86.7%) but a low specificity of 14.4 (DF) -19.3% (MS) (CI 95% of 9.7–25.6%). Sensitivity and specificity for each individual suture and synchondrosis is shown in Table 1.

### Sensitivity and specificity of suture assessment using CT

CT suture assessment shows a high sensitivity of 81.7 (DF) -85.1% (MS) (CI 95% of 76.2–89.3%) but a low specificity of 37.8 (DF) -40.4%(MS) (CI 95% of 48.5–75.8%). Individual suture and synchondrosal assessment is summarized in Table 2.

**Table 3:** Kappa values for the degree of interobserver agreement in CT and MRI

Suture/Synchondrosis	CT (ϗ -value)	MRI (ϗ -value)
S1	0.803	0.784
S2	0.936	0.649
S3	1	0.649
S4	0.53	0.714
S5	0.929	1
S6	0.934	1
S7	0.927	0.832
S8	0.934	0.832
S9	0.765	1
S10	0.817	1
S11	0.818	1
S12	1	0.869
SO	1	0.771
IS	1	0.894
SE	0.943	0.745

In dependence of observer, CT could not assess sutural state in 26 (MS)-31 (DF) of 455 sutures (6.8–8.1%).

### Interobserver variability

Regarding to the synchondroses of the cranial base an almost perfect agreement between the observers was shown for inter-sphenoidal synchondrosis (ϗ = 0.894) and a substantial agreement for the spheno-occipital (ϗ = 0.771) and spheno-ethmoidal synchondrosis (ϗ = 0.745). Depending on the examined sutures observer accordance in MRI reaches from 70.3% (CI 95% of 53–84.1%) to 94.9% (CI 95% of 82.7–99.4%). Spheno-frontal (ϗ = 0.832; 1), squamosal (ϗ = 0.832; 1), lambdoid (ϗ = 1), sagittal (ϗ = 1) and palatinal suture (ϗ = 0.869) showed an almost perfect agreement between the observers. Interfrontal (ϗ = 0.784), coronal (ϗ = 0.649) and sagittal suture (ϗ = 0.714) showed a substantial agreement (Table 3).

## Discussion

Synchondrosis and sutures are cranial growth centers that allow expansion of the brain and cranial cavity. An increasing body of evidence suggests that general head morphology in dogs may be substantially influenced by the function of these growth plates [15, 16]. A premature fusion of one or more cranial sutures may result in characteristic shortening of the cranial cavity and facial bones in brachycephalic dogs [15, 16, 20–23]. Ethical concerns as regards of breeding brachycephalic animals would arise if a pathological growth disorders would be the basis of their head morphology. The study of cranial sutures and synchondroses in dogs therefore merits more serious consideration. Non-invasive diagnostic imaging methods as MRI and CT are the primary imaging techniques used to evaluate the condition of the cranial sutures and synchondroses in children [10, 24]. They have also been used to investigate cranial growth centers in dogs [16]. The use of MRI and CT would permit the investigation of a large cohort of dogs, and would also allow monitoring the temporal behavior of a single suture in the same animal. However, a review of the veterinary literature revealed large gaps in knowledge about the morphology and ontogeny of cranial base synchondroses and cranial sutures and their assessment using imaging methods in dogs. This study was, therefore, designed to determine the sensitivity and specificity for both imaging methods using histological examination as a control.

For the assessment of synchondrosal status, both CT and MRI showed a good sensitivity and specificity. The large size of synchondroses, as well as the consistent change of signal (or density resp.) from cartilage to bone tissue account for this result. However, not all sutures could be assessed equally well. The spheno-ethmoidal synchondrosis has a lower sensitivity compared to spheno-occipital and intersphenoidal synchondrosis in both CT and MRI. The microanatomy of this synchondrosis differs from the others and is more difficult to define within the cranial base as it is not always straight but crescent-shaped [24]. The transition to the air filled nasal cavity blurs the contrast between the synchondrosis and the medullary cavity that allows good visualization of the other synchondroses.

Assessment of suture status was more difficult in both modalities. Although CT correctly identified a cranial suture as open (high sensitivity), it was less reliable in identifying a truly closed suture (low specificity). MRI revealed a moderate sensitivity and a low specificity for the assessment of sutural state. The low specificity for both modalities can be attributed to the small size of the examined structures, which can be hardly visible even on a prepared skull, especially in small dogs. Although CT and MRI scanning had a submillimeter resolution in this study, the small size of some sutures in very young and older dogs (micrometer range) complicates clear identification. Cranial sutures can be reliably studied using micro-CT of human skull specimens [25]. However, considering the limitations of the gantry size of the micro-CT, and extreme long scanning time, micro-CT is impractical for in-vivo radiologic evaluation of cranial sutures. Flat-panel-CT, which produces optic resolution between 200 and 250 μm allows visualization of calvarial sutures in vivo [26–28]. It is likely that sensitivity and specificity of suture assessment in dogs would be higher using this imaging technique.

The microstructure of the suture might also have an influence on its assessment. The spheno-frontal sutures and squamosal sutures were particularly difficult to examine. These two are overlapping sutures (sutura squamosa), with extremely thin sutural gaps, which are located laterally on the skull curvature, making an assessment even more difficult. Butt sutures (sagittal fissure, palatine fissure) do not overlap and have a wider sutural gap, which makes them easier to identify. Furthermore, the localization of the sagittal, and lambdoid sutures is marked by the external sagittal crest or the nuchal crest, respectively, which simplifies their identification in both imaging and histology.

Beyond size and morphology, the variability between the observers indicates that assessment of sutures is also experience dependent. Classifying the maturation stage of cranial sutures in children requires significant training and proficiency [29]. Although diagnostic methods to evaluate cranial suture ossification/maturation in human studies usually rely on Flat-panel-CT with higher resolutions, only images read by experienced viewers achieves good to excellent agreement compared to histologic slides [30]. Interrater variability for the assessment of cranial sutures in humans are consistent with the range of this study in dogs (0.67–0.84) [31].

In humans, the end point of cranial vault growth is determined upon fusion of the sutures in the third decade of life [5], but there can be considerable variability in closure rates [32]. It is interesting to note, that even in the old dogs the majority of the cranial sutures do not ossify. The interfrontal, coronal, sagittal and lambdoid sutures as well as the palatine fissure all provide strong evidence for maintained suture patency even in advanced age (up to 11 years). In our study we identified two types of syndesmoses, type A with loose connective tissue lying parallel to the suture line and consisting of a high amount of osteoblaststs at the sutural edges. Type B was considered to have dense connective tissue which is orientated in a ninty degree to the suture line and contained low numbers of osteoblasts. Exemplary findings of coronal, parieto-sphenoidal, parieto-interparietal and lamboid type B syndesmoses in older dogs (3 to 10 years) lead to the suggestion that this might be a form of inactive (functional closed) suture. The sutural space is reduced to ~ 100-200 μm but not bridged with bony tissue and remain as a syndesmosis. Cranial sutures in mammals do not necessarily fuse when growths stops or slows down suggesting that they have an additional role [18]. The transformation of the sutural structure allows flexibility and energy absorption in the skull bones and reduces the risk of skull fractures in mature [33], which is why they can remain in mature animals. The fact that the end-point of sutural development in the dog is not necessarily determined upon fusion demonstrates the impossibility to determine the physiological end point of bone growth in the suture on the basis of imaging techniques. A pathological craniosynostosis on the other hand might be diagnosed using CT or MRI.

Our study presents one important limitation. Histological sections without visible sutures were rated as fused. This finding might be based on the presence of a truly ossified suture, or because the suture was left out during sample cutting. Relatively simple at birth, the microarchitecture of many sutures gain complexity during growth. We observed a three-dimensional structure and their internal course must not necessarily correspond to the visible suture on the surface. The validity of our results might have increased further if the whole excised bone/suture sample would have been fully sliced, but we refrained from the examination of the whole suture course as this process is extremely time consuming.

## Conclusion

Conventional imaging techniques are very useful to assess the open or closed status of synchondroses within the cranial base. Sutural closure is difficult to diagnose in MRI and CT. Assessment is dependent on observer experience. Histologic examination remains the gold standard for suture assessment.

## Methods

The aim of the present study was, therefore, to compare imaging findings with histological preparations in order to determine the sensitivity and specificity for conventional clinical CT and MRI in the assessment of the open or closed status of cranial synchondroses and sutures in dogs.

### Animals

The heads of 40 dogs of different breeds (24 mesaticephalic- and 16 brachycephalic dogs), were collected from the Department of Veterinary Clinical Sciences, Clinic for Small Animals and from the Clinic for Gynecology, Andrology and Obstetrics of the Justus-Liebig-University, Giessen (Table 4). The dogs were euthanized or died due to diseases unrelated to the skull and central nervous system. Age and sex was recorded. Written consent was obtained from all owners that donated their animals for the study and actual dogs remained anonymous.

**Table 4:** Breed, age and sex of mesati- and brachycephalic dog breeds included in the study

Number	Mesaticephalic dog breeds	Age (days)	Sex	Number	Brachycephalic dog breeds	Age (days)	Sex
1	German Shepherd dog	0	F	1	Cavalier King Charles spaniel	0	F
2	German Shepherd dog	0	M	2	Cavalier King Charles spaniel	0	F
3	German Shepherd dog	0	F	3	Chihuahua	0	M
4	Pomeranian	0	M	4	American bulldog	0	F
5	Dachshund	0	M	5	American bulldog	0	F
6	St. Bernhard	1	F	6	Cavalier King Charles spaniel	1	M
7	Irish Wolfhound	3	F	7	French bulldog	2	F
8	Pyrenean Shepherd dog	3	F	8	French bulldog	2	f
9	German Shepherd dog	7	F	9	Pug	56	M
10	Golden Retriever	28	F	10	Chihuahua	56	M
11	Dachshund	35	M	11	Pug	84	M
12	West Highland White terrier	35	F	12	Chihuahua	84	F
13	Pomeranian	70	M	13	Mixed breed	140	F
14	Collie	84	F	14	Prague Ratter	224	M
15	Jack Russel terrier	84	M	15	Shih Tzu	280	M
16	Beagle	98	F	16	Pug	392	M
17	Mix breed	112	M				
18	German Shepherd dog	112	M				
19	Mix breed	112	F				
20	German Shepherd dog	126	M				
21	Shiba Inu	168	M				
22	Mix breed	1092	F				
23	German Shepherd dog	1092	M				
24	Spanish greyhound	3642	M				

### Imaging techniques

Post mortem MRI of the head was obtained using a high field scanner ^(^Gyroscan Intera, 1.0 T, Phillips, Hamburg, Germany). Dorsal, sagittal, and transvers T2-weighted (W) images with a 2 mm slice thickness and a 0.2 mm slice interval were acquired (T2-Turbospin echo, echo time (TE) 120 ms, repetition time (TR): 2900 ms. T1-FFE weighted dorsal and transversal images with a slice thickness of 1 mm were obtained (TR 25 ms TE 6.9 ms). Field of view was 120 × 120 mm in small dogs and 210 × 210 mm in large dogs. Matrix was 288 × 288 in small dogs and 384 × 384 in large dogs leading to a pixel size between 0.625 × 0.625 mm and 0.54 × 0.54 mm.

Post mortem CT examination was performed using a sixteen slice CT scanner ^(^Philips Brilliance 16, Phillips, Hamburg, Germany^)^. Transvers images with a slice thickness of 0.8 mm were obtained using 120 kV, 321 mA and a field of view of 133 mm. Data were processed using a bone algorithm (window width 2500 and window level 500).

### Image analysis

**Fig. 5:**
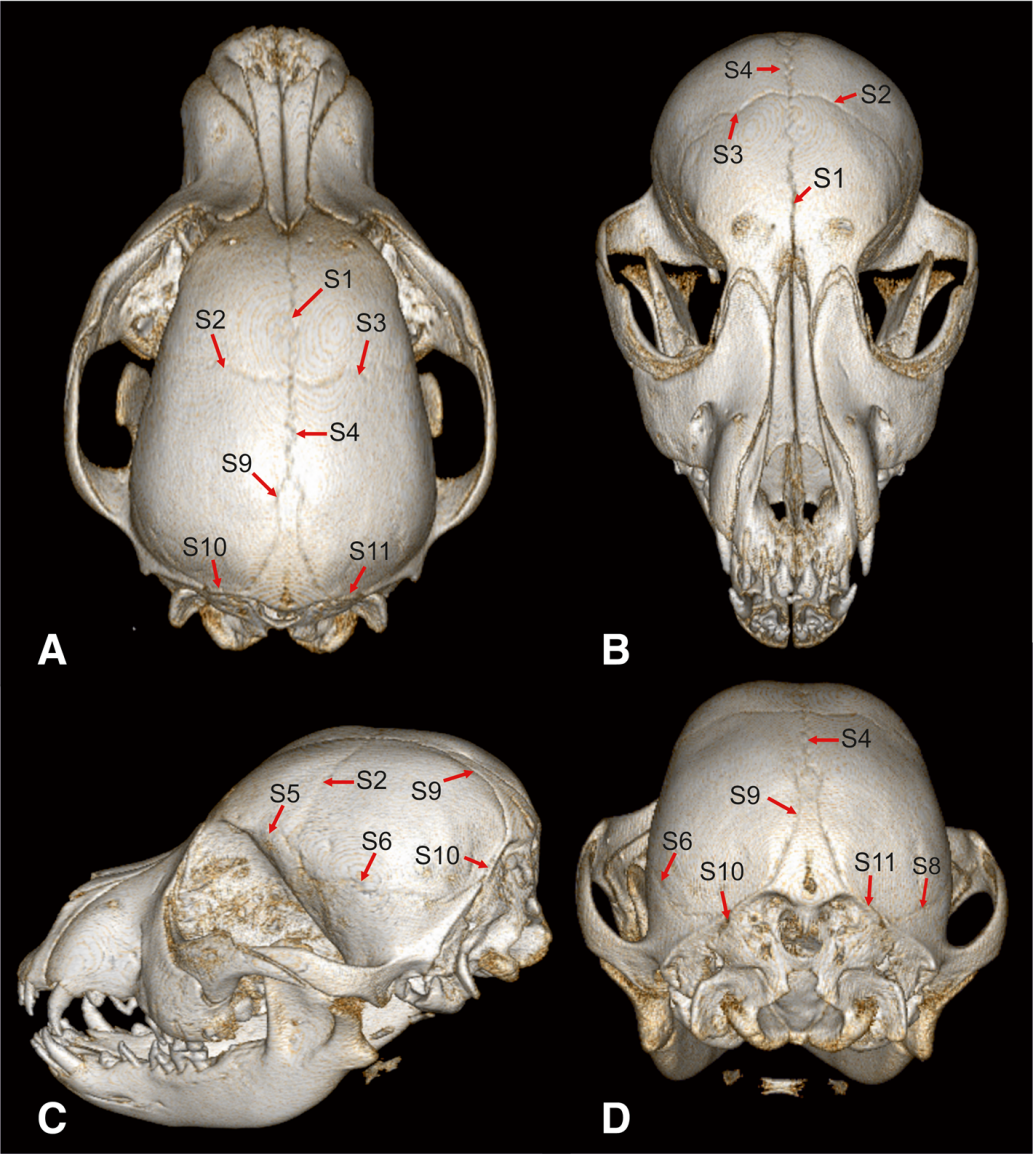
Overview of cranial sutures in the skull of a 4.5-months-old German shepherd dog. Three-dimensional volumetric reconstructions of the CT data of the skull of a 4.5-months-old German shepherd dog in dorsal view (**a**), frontal view (**b**), left lateral view (**c**) and caudal view (**d**). S1 = interfrontal (metopic) suture, S2/3 = left and right fronto-parietal (coronal) suture, S4 = sagittal suture, S5 = left spheno-frontal suture, S6/8 = left and right squamosal suture, S9 = parieto-interparietal suture, S10/11 = left and right lambdoid suture

**Fig. 6:**
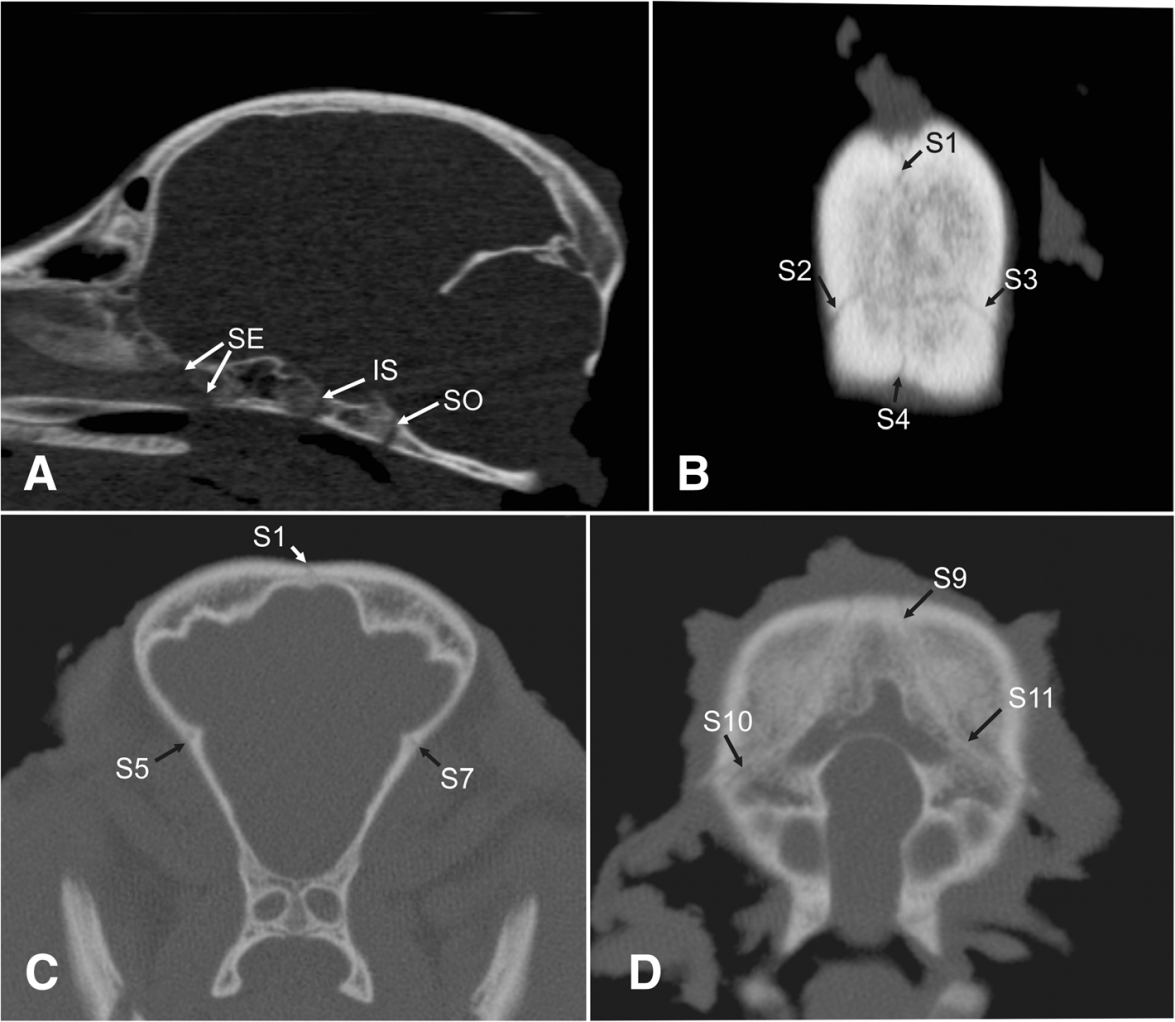
Representative CT images of a 4.5 months old German shepherd dog. Computed tomography images of a 4.5-months-old German shepherd dog skull in reconstructed midsaggital view (**a**), reconstructed dorsal view (**b**), transverse rostral view (**c**) and transverse caudal view (**d**). S1 = interfrontal suture, S2/3 = left and right fronto-parietal suture, S4 = sagittal suture, S5/7 = left and right spheno-frontal sutures, S9 = parieto-interparietal suture, S10/11 left and right lambdoid suture. Cranial base: spheno-ethmoidal synchondrosis (SE), intersphenoidal synchondrosis (IS) and spheno-occipital synchondrosis (SO)

**Fig. 7:**
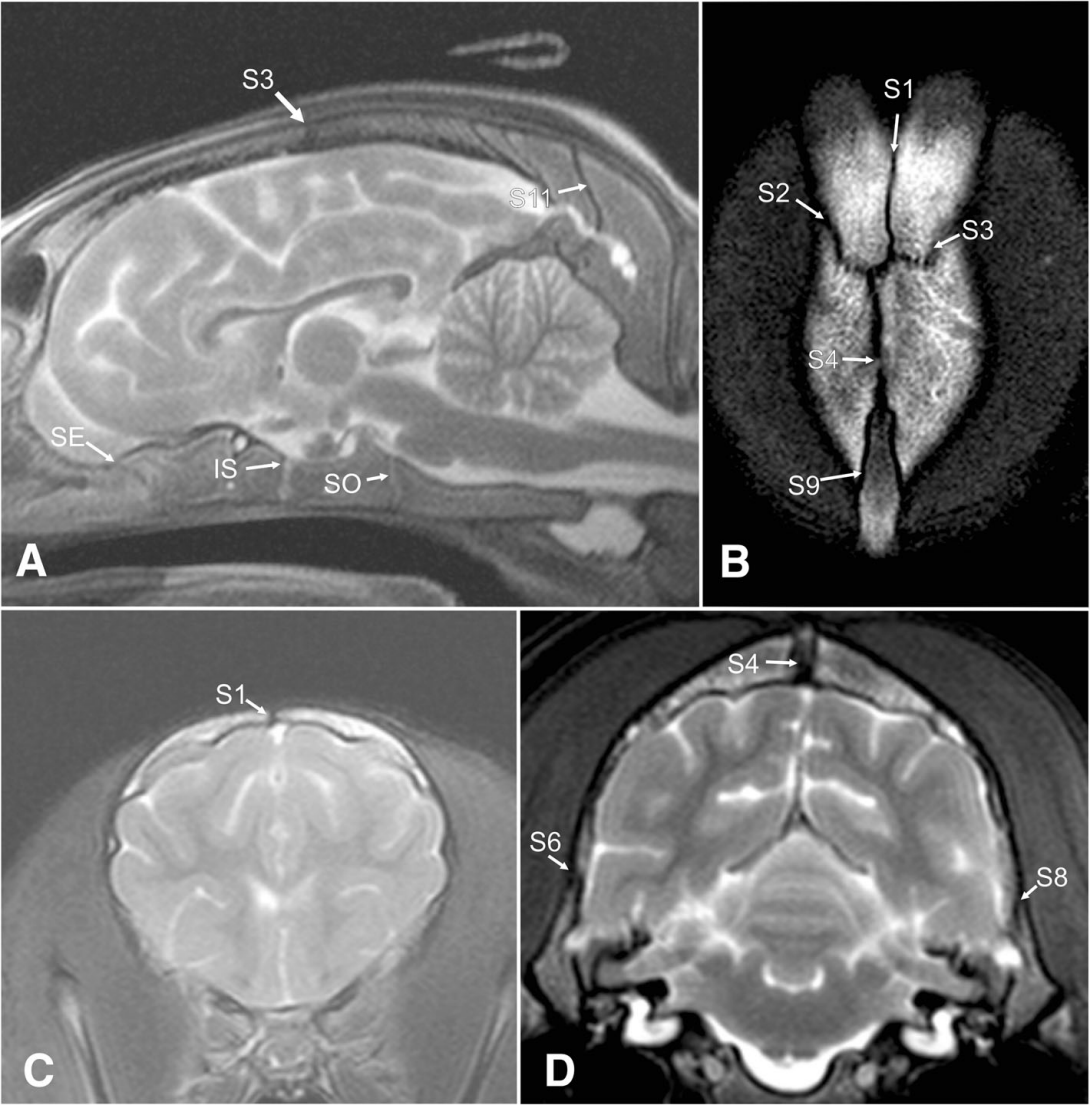
Representative T2 weighted MRI of a 4.5 months old German shepherd dog head. Magnetic resonance images of a 4.5-months-old German shepherd dog skull in in a midsagittal (**a**), dorsal (**b**), and transverse view (**c**, **d**). S1 = interfrontal suture, S2/3 = left and right fronto-parietal suture, S4 = sagittal suture, S6/8 = left and right squamosal suture, S9 = parieto-interparietal suture, Cranial base: spheno-ethmoidal synchondrosis (SE), intersphenoidal synchondrosis (IS) and spheno-occipital synchondrosis (SO)

**Fig. 8:**
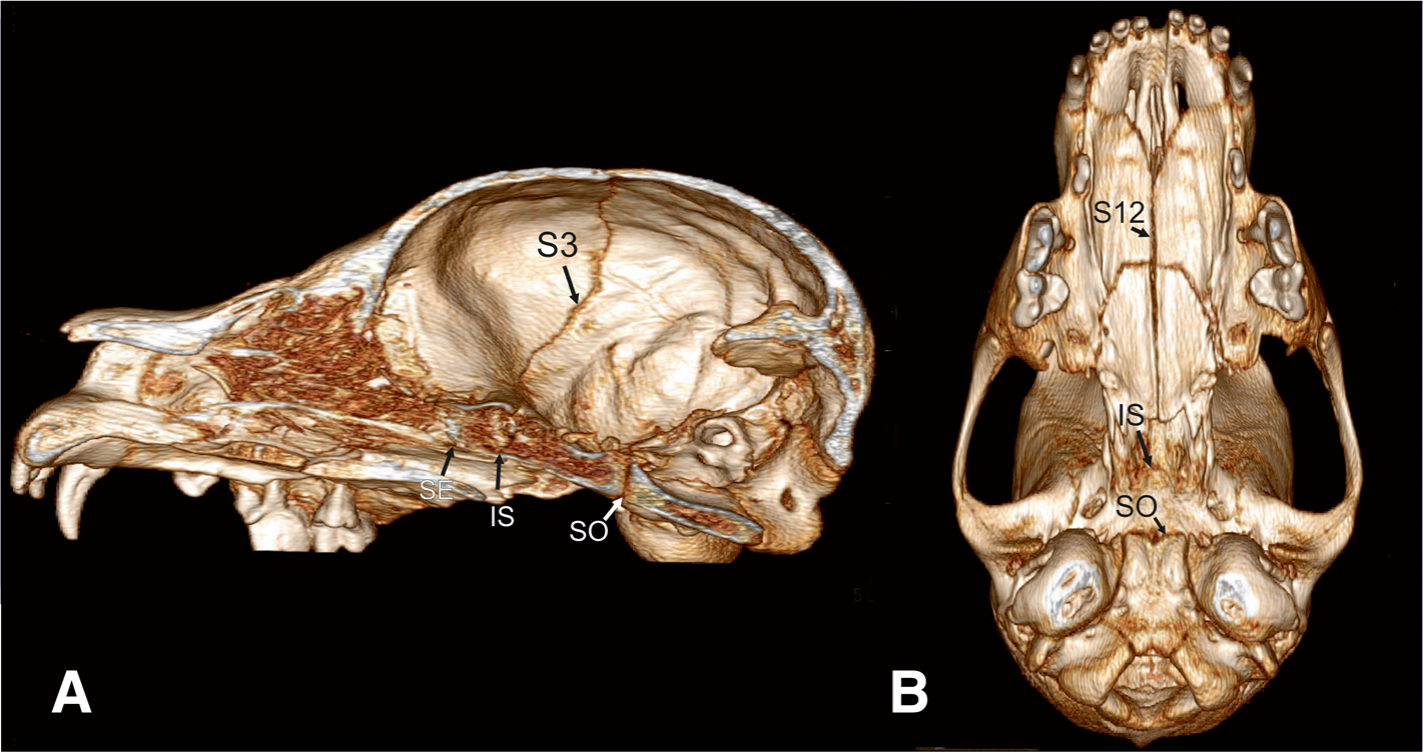
Overview of cranial sutures and synchondroses in the skull of a 4.5 months old German shepherd dog. 3D reconstruction of the skull based on computed tomography images of a 4.5-months-old German shepherd dog skull in midsaggital view (**a**) and ventral view (**b**). S3 = right fronto-parietal suture, S12 = median palatine fissure. Cranial base: spheno-ethmoidal synchondrosis (SE), intersphenoidal synchondrosis (IS) and spheno-occipital synchondrosis (SO)

All images were retrieved from the relevant picture archiving system and evaluated by a board certified neurologist (MJS) and a doctoral student (DF). The experiments were performed using anonymized and randomized image data sets. The observers were blinded to age and breed of the dog. Studies were evaluated with open source DICOM viewing software and window levels, window widths, and magnification were adjusted as needed in order to optimize visualization of bone. The following cranial growth centers were examined: Interfrontal (metopic) suture (Fig. 5a and b; Fig. 6b and c; Fig. 7b and c: S1), fronto-parietal (coronal) sutures (Fig. 5a, b and c; Fig. 8a; Fig. 6b; Fig. 7a and b: S2/3), sagittal suture (Fig. 5a and d; Fig. 6b; Fig. 7a and d: S4), spheno-frontal suture (Fig. 5c; Fig. 6d: S5/7), squamosal suture (Fig. 5c and d; Fig. 7d: S6/8), parieto-interparietal suture (Fig. 5a,c,d; Fig. 6d; Fig. 7a and b: S9), lambdoid suture (Fig. 5a,c,d; Fig. 6d; Fig. 7b: S10/11), median palatine fissure (Fig. 8b: S12), spheno-ethmoidal synchondrosis (Fig. 8a and b; Fig. 6a: SE), intersphenoidal synchondrosis (Fig. 8a,b; Fig. 6a: IS), and spheno-occipital synchondrosis (Fig. 8a and b; Fig. 6a: SO). All interpretations were made by the two observers independently to determine interobserver variability. Results of the image interpretation and histological findings were compared.

### Assessment of synchondrosis and suture status in CT

In CT, an open synchondrosis or suture was defined as a hypodense zone between well-defined hyperdense borders of the calvaria or cranial base bones (Fig. 6). A closed suture or synchondrosis was defined as a continuous hyperdense bone, without interruption by a hypodense structure. Partial closure was defined as a non-continuous hypodense bone and the presence of bone-isointense bridges within this hypointense bone structure.

### Assessment of synchondrosis and suture status in MRI

In MRI, an open suture was defined as a hypointense signal interruption of the hyperintense calvarial bone marrow signal in T2 weighted images. An open synchondrosis was defined based on the presence of a broad hyperintense signal zone (cartilage) with well defined, hypointense borders (endplates) (Fig. 7). A closed suture was defined as a lack of a hypointense signal within the hyperintense bony structures. A closed synchondrosis was defined based on the obliteration of the synchondrosis, replacement of cartilage and development of a continuous isointense signal from the bone marrow cavity. Partial closure was defined as bony bridges within the suture or synchondrosis, visible as a partial hypointense signal of a suture within the hyperintense bone, or as a hypointense signal within the hyperintense cartilage signal of the synchondrosis, with a narrowing of the synchondrosal cartilage.

### Histological sample preparation

After postmortem scanning soft tissues were carefully removed from the skull and the whole head was immersion fixed in 10% neutral buffered formalin. A standardized procedure was devised to cut out the sutures and synchondroses from each head using a diamond coated sawblade (Proxxon Micromot System^)^. Suture morphology was assessed as precisely as possible in a plane of sectioning perpendicular to the individual suture orientation [18]. Not all sutures could be macroscopically identified in all skull specimens. These individual sutures were not included in the calculation of sensitivity and specificity. Samples were washed with 1% phosphate buffered saline and decalcified over 4 weeks with Ethane-(1,2-diyldinitrilo) tetraacetic acid with a pH = 8. After decalcification, specimens were embedded in paraffin (Parablast, Sherwood^)^. Three serial sections of 5 μm were stained using Masson-Goldner protocol in order to visualize bone, cartilage and fibrous connective tissue. The stain produces blue or green collagen and deep red mineralized bone.

### Histomorphological analysis of the sutures

Histological slides were evaluated for structural characteristics indicating an open or closed state of the sutures. Two types of open sutures were defined according to the type of connective tissue which dominated the sutural space. The loose connective tissue type lying parallel to the suture line was defined as open suture type A and contained a high amount of osteoblasts (> 50 in a mean of 3 field of views in a magnification of 40 (FOV)) and fibroblasts (> 20 in 3 FOV)) (Fig. 4a.). The dense connective tissue which is orientated in a ninty degree to the suture line was characterized as open type B, it contained low numbers of osteoblasts (< 10 in 3 FOV) and fibroblasts (< 20 in 3 FOV) (Fig. 4b.). Partly closed sutures describes bony bridges within the suture. In some cases no suture could be identified in the collected sample.

### Histomorphological analysis of the synchondroses

The synchondroses were defined as open when there was a continuous zone of cartilage between the bones (Fig. 1), and as closed when there was no cartilage visible and there was bony tissue showing a continuing medullary cavity. Partly synostosis was defined as non-continouous cartilage next to bony tissue between the periostal borders.

Each section was histologically analyzed in a two-step procedure. First, the existence or non-existence of a fibrous tissue or cartilage within the synchondrosis/suture was determined by use of a histological overview (40x magnification). Non-existence of cartilage or fibrous tissue was evaluated as a closed synchondrosis/suture. Furthermore, it was determined whether fibrous- or cartilage tissue was present throughout the suture/ synchondrosis, or whether bony bridges were present. These data was used to assign each synchondrosis/suture to one of the following categories: 1) open synchondrosis/ suture; 2) partly closed synchondrosis /suture; 3) closed synchondrosis/suture (Figs. 1, 2). Morphological analysis of sutural appearance, collagen structure within the sutural space and amount of osteoblasts, fibroblasts and blood vessels was also performed.

### Statistical analysis

All statistical analyses were performed using the statistical software package BMDP. Sutures classified as open in MRI and CT were compared to type A syndesmoses. Sutures classified as closed in MRI and CT were compared to type B syndesmoses indicating an inactive or functionally closed condition. As the partially closed state does not allow longitudinal extension partially synostosis was also considered as a closed synchondrosis or suture and summed up for statistical analyses. An exact-fisher test was performed on statistical data to compare different suture assessment techniques of histological examination, MRI and CT. A high sensitivity means, in this regard that a synchondrosis or suture, which was assessed as open in imaging was also assessed as open in the histological examination. A high specificity means that the closed status of a synchondrosis or suture in MRI and CT was confirmed by histological evaluation. Sensitivity and specificity in MRI and CT were defined as high (> 80), moderate (65–79%) or low (< 64%). Interobserver variability was obtained using the kappa coefficient. A kappa value < 0.2 implies slight agreement, 0.21–0.4 fair agreement, 0.41–0.6 moderate agreement, 0.61–0.8 substantial agreement and > 0.81 almost perfect agreement.

## Data Availability

Data and materials are available from the corresponding author on reasonable request.

## Abbreviations

Cl 95%: Confidence level of 95%
CT: Computed tomography
DF: Daniela Farke
F: Female
FOV: Field of view
IS: Intersphenoidal synchondrosis
M: Male
MJS: Martin Jürgen Schmidt
MRI: Magnetic resonance imaging
S1: Interfrontal suture (metopic suture)
S10: Left lambdoid suture
S11: Right lambdoid suture
S12: Medial palatine fissure
S2: Left fronto-parietal suture (coronal suture)
S3: Right fronto-parietal suture (coronal suture)
S4: Saggital suture
S5: Left spheno-frontal suture
S6: Left squamosal suture
S7: Right spheno-frontal suture
S8: Right squamosal suture
S9: Parieto-interparietal suture
SE: Spheno-ethmoidal synchondrosis
SO: Spheno-occipital synchondrosis
μm: micrometer
ϗ: kappa value
